# A Clinician’s Perspective on the Accuracy of the Shade Determination of Dental Ceramics—A Systematic Review

**DOI:** 10.3390/jpm14030252

**Published:** 2024-02-27

**Authors:** Katarzyna Dudkiewicz, Szymon Łacinik, Maciej Jedliński, Joanna Janiszewska-Olszowska, Katarzyna Grocholewicz

**Affiliations:** 1 Student Scientific Society at the Department of Interdisciplinary Dentistry, Pomeranian Medical University in Szczecin, 70-111 Szczecin, Poland; 63632@student.pum.edu.pl (K.D.); 62961@student.pum.edu.pl (S.Ł.); 2 Department of Interdisciplinary Dentistry, Pomeranian Medical University in Szczecin, 70-111 Szczecin, Poland

**Keywords:** dental ceramics, color determination, shade estimation, shade guide, spectrophotometer, colorimetry, prosthodontics

## Abstract

Background: No systematic review or meta-analysis has been identified that provides a clinician’s perspective on the shade selection process for ceramic restorations. The aim of the present systematic review is to find and systematize the available knowledge by referring to the methods to assess the color of dental ceramics. Methods: The final search was performed on 10 December 2023 in six search engines. The keywords used in the search strategy were as follows: (“color matching” OR “shade matching” OR “color measurement” AND “porcelain” OR “dental ceramics”) AND “dentistry” AND “accuracy”. Results: The search strategy identified 139 potential articles. After the screening process, sixteen articles were included in the review. Conclusions: In conclusion, the most common method, the visual method, has lower accuracy and repeatability. Devices like spectrophotometers and colorimeters provide precise, repeatable, and objective measurements, but fail to be widely applied in everyday clinical practice. Clinicians should not rely solely on their senses for shade determination, but should turn to quantitative methods. Colorimetric devices connected to mobile applications are an interesting and useful tool. Software and apps based on artificial intelligence are emerging as promising tools, but they require further research.

## 1. Background

Shade determination is a crucial treatment step in prosthetic dentistry. It can be defined as a visual perception within the visible spectrum of electromagnetic radiation. To standardize the shade determination/color selection process, a committee was established to create international standards for color description. CIE L*a*b* is a color notation system defined by the International Commission on Illumination in 1976 and it is constantly amended. This system allows each color to be described with a numerical value of L, a and b, whereas L stands for luminance, while a and b are chromaticity coordinates. It is a commonly used system in dentistry, both in vitro and in vivo [1]. Nowadays, color measuring dental devices are based almost entirely on the CIELAB color space for color measurements [2]. The color of natural teeth is not uniform in all regions. Usually, it can be divided into three parts: gingival, middle, and incisal. All of these parts present slightly different shades and opacity [3]. In the cervical part, dentine is thicker and enamel is thinner, making the tooth shade more opaque and darker. Incisally, dentine is thinner and enamel is thicker; hence, the tooth shade is more translucent and brighter. Color matching is subjective and relies on numerous factors. The subjective sense of color can alter depending on various conditions associated with the point of observation, such as the light source [4,5], background color [6] and occlusal factors [7], and the color and opacity of the tooth on which the crown is placed [8]. It was evidenced that shade determination is also biased by operator-related factors such as gender, age, experience, and tiredness [9]. The methods to assess or measure the color or shade of dental structures or reconstructions comprise a visual subjective assessment and instrumental assessment. A visual subjective assessment is based on the clinician simply comparing the patient’s teeth to an available shade guide, such as VITA 3D Master [10], which has already proved itself clinically. The latter methods allow an objective measurement but require equipment, such as spectrophotometers (SPM), colorimeters (CCM), spectroradiometers (SRM), photo cameras, backpropagation neural networks, or smartphone cameras with dedicated software [9]. A spectrophotometer is a device performing full-spectrum colorimetric measurements, making it superior to a colorimeter, which assesses color through three filters: red, green, and blue. A spectroradiometer is another tool that measures intensity and radiance using light intensity calibration; it operates in the visible and UV ranges of visible wavelengths from 300 to 700 nm [11]. Another device is a high-profile optic camera found in photographic devices or mobile phones. Mobile phone cameras can compete with traditional photo cameras due to their advanced optic systems [12]. They can have a built-in flash or separately bought ring lights. An analysis of such a photograph with the appropriate software can also be a way to determine the shade [13]. Another form of color determination is using backpropagation neural networks based on large number of teeth creating a database. It is a relatively new method based on implementing information, such as photos or data from spectrophotometers, to a neural network and receiving an output [14]. Shade selection is a basic procedure aimed at personalizing dental treatment and obtaining the optimum results in objective terms for a particular patient. It is especially important in expensive and time-consuming procedures, such as ceramic restorations [15]. For such procedures, everything must be performed to ensure success in every detail, above all in visual aesthetics [16]. While one can find many reports on composite colorimetry, when it comes to ceramic color matching, the topic does not seem to be as popular, especially when it comes to implementing solutions from the academic field into everyday clinical practice [17]. Although a systematic review was recently published regarding the color difference for shade determination with visual and instrumental methods [18], no systematic review or meta-analysis has been identified that provides a clinician’s perspective on the shade selection process for ceramic restorations. The present systematic review aims to find and systematize the available knowledge by referring to the methods to assess the color of dental ceramics.

## 2. Materials and Methods

### 2.1. Search Strategy

**Figure 1:**
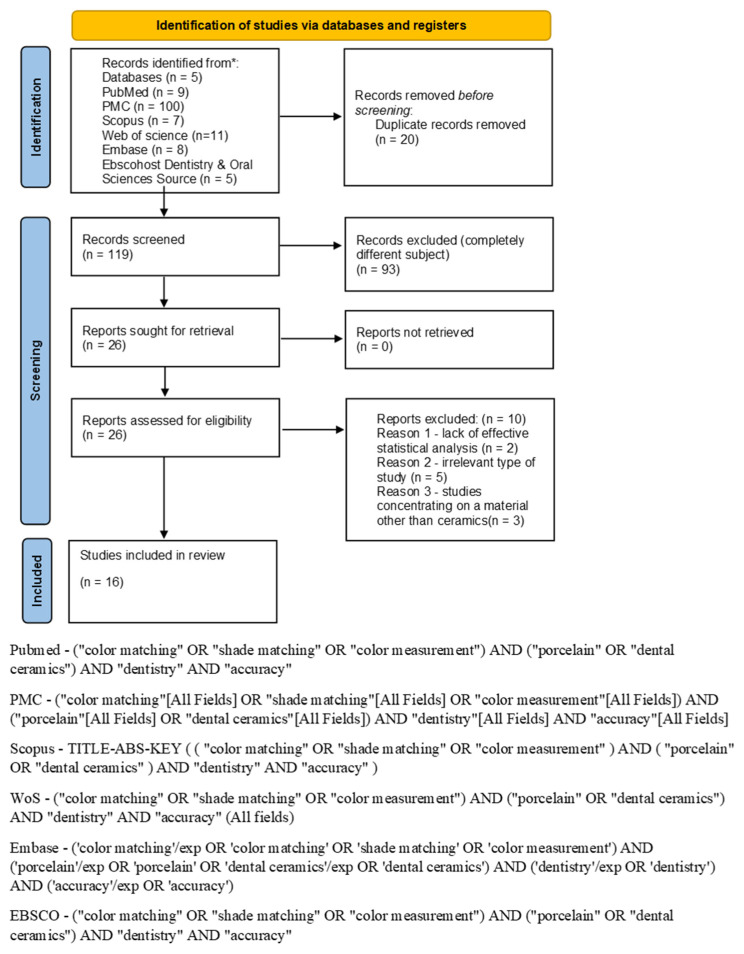

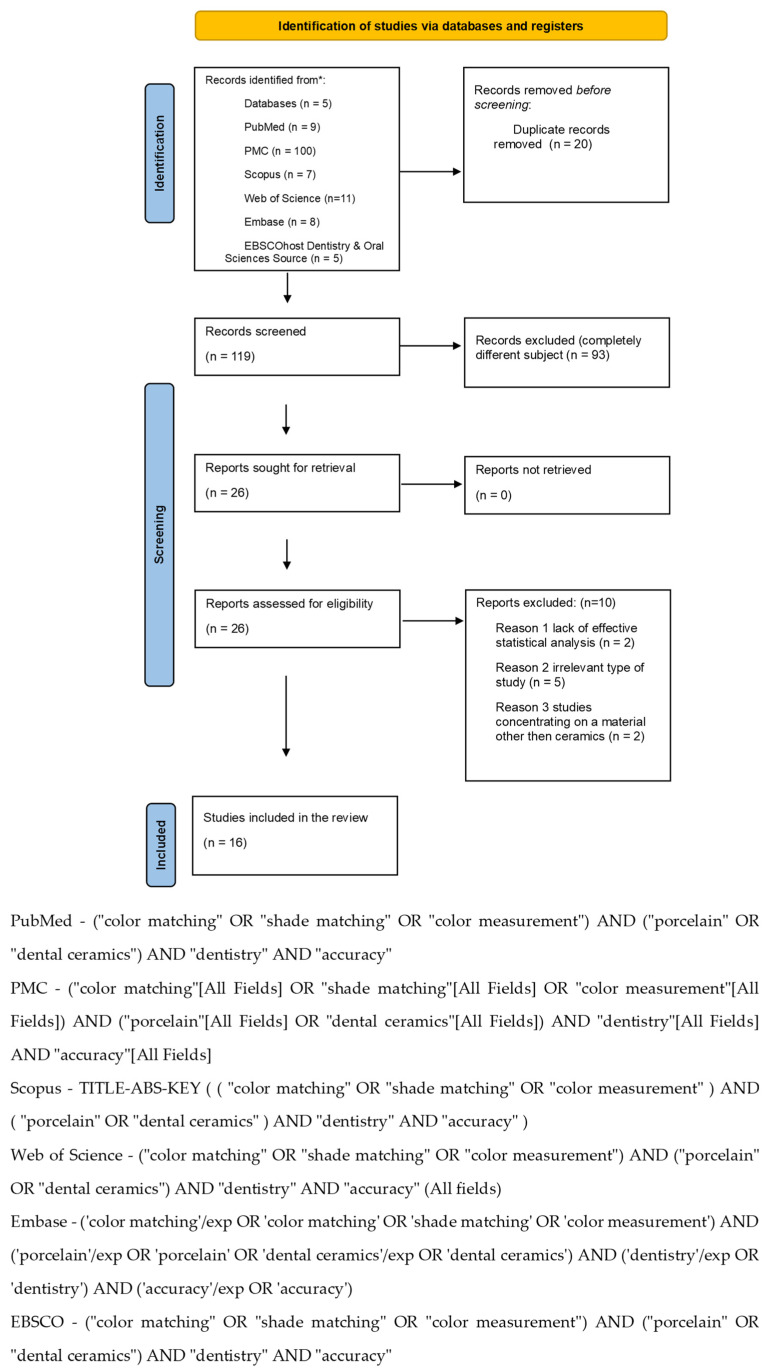
PRISMA 2020 flow diagram.

The review process was performed in compliance with the amended PRISMA statement [19], the PRISMA 2020 reporting guidelines ([20], Supplementary Materials S1) and the guidelines from the Cochrane Handbook for Systematic Reviews of Interventions [21]. The PubMed, PubMed Central, Web of Science, Scopus, Embase, EBSCOhost Dentistry & Oral Sciences Source search engines were applied in this search. The final search strategy was determined through several pre-searches of popular tags and MeSH terms in the topic studied. The first search was performed on 22 November. The final search was performed on 10 December 2023. The keywords used in the search strategy were as follows: (“color matching” OR “shade matching” OR “color measurement” AND “porcelain” OR “dental ceramics”) AND “dentistry” AND “accuracy”. The PRISMA 2020 flow diagram (Figure 1) demonstrates the exact search string in every engine applied. The framework of this systematic review according to PICO(S) [22] was as follows: population—dental prosthetic clinicians, dental technicians, dental students, and porcelain specimens; comparison—difference in accuracy of the porcelain shade determination by humans and color measurement devices; outcomes—ΔE according to CIELAB, accuracy and repeatability of shade selection according to different shade scales; and studies—in vitro studies and prospective clinical trials. The PICO(S) questions were as follows: Are the color measurement devices significantly more precise then dental clinicians? Does the use of objective color measurement methods carry significant improvements in clinical practice over standard subjective methods? The included articles discuss the determination of dental ceramics’ color with different methods. For all data and study characteristics evaluations, Zotero software 6.0.30. was used (Corporation for Digital Scholarship, Vienna, VA, USA).

### 2.2. Inclusion Criteria

The following inclusion criteria were applied for this systematic review: (a) prospective and retrospective clinical studies on dental ceramic determination, (b) in vitro studies, and (c) deep learning applications.

The exclusion criteria were the following: (a) case reports, (b) book chapters, (c) descriptions of techniques, (d) research without quantitative evaluations, and (e) records unrelated to the topic.

There were no language and no publication time restrictions applied.

### 2.3. Data Extraction

After retrieving the results from the search engines to create a database, the duplicates were removed. The literature was selected following the inclusion criteria by reading the titles and abstracts by two authors (KD and SŁ). Whenever a disagreement occurred, it was resolved by the study supervisor’s (MJ) decision. The full text of each record related to the topic was sought and read in order to determine if it was suitable for inclusion. Data were sought regarding the dental ceramics’ color determination. The authors extracted the values used in most of the studies and thus could be compared. Cohen’s K coefficient for the agreement between the authors in study selection was high and yielded 0.96. Authorship, year of publication, type of each eligible study, and main results were extracted by one author (KD) and examined by another author (MJ). The protocol was not registered. Data that were sought were indices for color determination accuracy.

### 2.4. Quality Assessment

According to the PRISMA statement [20], the assessment of methodological quality indicates the strength of the evidence provided by the study, as methodological flaws can cause bias. In order to perform a proper quality assessment, study-type-specific risk of bias tools were introduced in this study. Due to the fact that two types of studies, cross-sectional and in vitro studies can be individualized, two different tools were applied. For cross-sectional studies, a Newcastle–Ottawa Scale was applied. The scale for cross-sectional studies consists of three main categories: selection (5 questions and 5 stars maximum), comparability (1 question and 2 stars maximum) and outcome (2 questions and 3 stars maximum) [23]. The quality of the study is indicated by a high score. For in vitro studies, the QUIN assessment tool was used. This tool consists of twelve different criteria that thoroughly assess the quality of the study. Two authors used the subsequent scoring system: (i) adequately specified (2-1 points); (ii) not specified (0 points); and not applicable (exclusion from calculation). Then, all the points are summed. The overall score for the given research was calculated to classify the risk of bias. A study that receives 70% or more is considered a low risk of bias study, 50–70% = a medium risk of bias, while the studies which receive 50% or less points = a high risk of bias study [24]. The Jadad Scale is a tool for evaluating the quality of randomized clinical trials (RCTs). It consists of five questions. The first three questions, which deal with the randomization of subjects, the blinding of patients and operators, and the proportion of subjects lost to follow-up, are answered with a binary response (yes earns 1 point, no earns 0 points). Depending on the adequacy of the randomization and double blinding, one point is either added or subtracted from the total score of the first three questions. This results in a final score that can range from 0 to 5, with 0 indicating a low-quality study and 5 indicating the highest quality. A study is deemed to be of good quality if it achieves a score of 3 or more [25].

## 3. Results

### 3.1. Search Results

The search process is described in detail in Figure 1.

The search strategy identified 139 potential articles from all six search engines. After the removal of 20 duplicates, 119 articles were analyzed. All articles were identifiable. Subsequently, 93 articles were excluded because they were not relevant to the topic. Of the 26 remaining articles, 10 were excluded because they were not relevant to the full-text analysis. The remaining 16 articles were included in the review and are presented in the Table 1.

Overall, including all in vitro studies mentioned in the presented systematic review, 104 full-ceramic tabs and 123 metal–porcelain crowns were examined. Devices included in all studies consist of five spectrophotometers, one colorimeter, two neural networks, and one photo camera with external software. The clinical studies examined five full-ceramic tabs, seventy-three metal–porcelain crowns, and nine natural teeth. One spectrophotometer, one colorimeter, and four porcelain shade guides were used.

All objective measurements are described in delta E, which stands for the difference according to CIELAB, while subjective measurements were according to the shade scale of VITA^®^.

There are very few clinical trials that have used advanced objective methods to assess color, and the scientific evidence is mostly based on in vitro studies.

In each of the studies mentioned, qualitative methods are far less effective than quantitative methods. Factors that may influence clinicians’ self-selection of color are outlined. In studies indicating the use of new software solutions to accelerate and facilitate the shade determination process, researchers try to demonstrate the compatibility of the new solutions’ results with a spectrophotometer or other classical device.

### 3.2. Quality Assessment

The results of the risk of bias assessment are presented in Table 2, Table 3 and Table 4, consequently.

The quality of the included cross-sectional studies was considered high in case of two studies [34,39], medium in the case of two studies [27,37], and low the in case of one study [29]. Only in one study was a proper sample size adjustment performed [39] and only in two studies were the coexisting factors hindering the study analyzed. Four of five cross-sectional studies provided in depth analyses of subjects [27,34,37,39]. The quality of the included in vitro studies was considered medium in the case of seven studies [26,28,31,32,36,38,41] and high in the case of two studies [30,35]. The most frequent shortcomings that occurred in included studies are i.e., a lack of sample size calculation, proper randomization, proper description of outcome assessors, and lack of blinding of research personnel. The only included randomized clinical trial met all the criteria foreseen in the Jadad scale, and thus can be considered a high-quality study [40]. Importantly, there is not yet a widely used scale for analyzing the quality of neural networks, and so quality assessment has been left for the original type of study.

## 4. Discussion

This systematic review aimed to establish evidence of possible factors affecting color determination in prosthetic dental restorations and the clinical effectiveness of different shade determination methods.

### 4.1. Subjective Color/Shade Determination

Color/shade matching using only visual methods is unreliable enough to achieve acceptable and repeatable results. Research shows that aspects such as sex [37], clinical experience [42], tiredness [34], or even personality type [34] or ocular dominance can affect the final perception of color. Thus, the intra- and inter-examiner compliance in this method is low. Nonetheless, even when a given clinician is predisposed and more sensitive to distinguishing color/shade than others, visual color matching is less effective than using dedicated devices [42]. Therefore, shade matching might be problematic, especially for clinicians with less experience [11].

Ceramics are widely used in prosthetics because they are known for their high aesthetics and chemical stability. Ceramics contain two phases: crystalline, which provides strength, and glassy, which provides fragility and transparency.

Dental ceramics can be categorized by the type of restoration:

- Traditional dental ceramics—silica-based ceramics;
- Glass–Ceramics—leucite-reinforced feldspathic ceramics, fluormica glass–ceramics, lithium disilicate ceramics;
- High-strength core ceramics—glass-infiltrated ceramics, metal-oxide based ceramics [43].

Due to such a wide variety, there can be no question that one shade guide will be suitable for different types of ceramics. Shade guides are a widely used tool in prosthetics because they are a universally recognized benchmark and are not expensive. They consist of porcelain tabs placed on a handle with a shade name. Shade guides mentioned in research are Vita Classical, Vita 3D Master, Vita Lumin Vacuum, and Ivoclar Chromascop [27,29,32,36,37,39,44]. All shade guides are meant for the same purpose. However, some of them are more accurate than others. Research shows that Vita 3D Master provides the most repeatable results in color matching [27]. Moreover, it can be helpful, especially for clinicians with less experience or who do not specialize in prosthodontics [44]. The better performance of Vita 3D Master might result from a few aspects. It consists of more tabs: 26 compared to 16 in Vita Classical. Shade matching using Vita 3D Master requires three-step shade determination (brightness, intensity, and shade), while Vita Classical has only one step. Their disadvantages, however, are the bias of the measurement author and the need to learn to distinguish nuances in color selection. This means that such selection is always qualitative and thus debatable, rather than based on objective data [45]. On the other hand, it should be emphasized that it is the most popular, least expensive to implement, and, unfortunately, mistakenly deemed as the simplest to learn of all the methods mentioned in the review.

### 4.2. Objective Color/Shade Determination

The available literature states that current colorimetric and spectrophotometric tools are reliable and can perform repeatable results [46]. The comparison of this type of device was initially mentioned by Seghi [26] at the very vegging of device-supported color matching compared to visual methods [26,28,29,46]. Spectrophotometers, such as Vita EasyShade V [36,38,39] or Olympus CrystalEye [25], proved helpful in everyday clinical practice and significantly improved performance, especially in prosthodontics.

Spectrophotometers can provide reliable results and objective measurements according to CIE L*a*b*—SpectroShade 2.20—or according to shade guides—VITA EasyShade V [47].

However, spectrophotometers and other colorimetric devices serve only one specific purpose, which is shade matching. Therefore, some clinicians, especially those not specializing in prosthodontics, might prefer to avoid investing in such a device. They are an additional expense and require an inquiry into color theory, such as the CIE L*a*b* system. Moreover, they require a technician capable of properly interpreting the measurement results and preparing the appropriate ceramics. For this reason, despite their exceptional performance and accuracy, the presented devices are not widely used among dental professionals [47].

One is able to perceive a constant development in regards to objective shade determination methods. Recently, a new type of colorimetric device was found. The device itself is more compact than a regular colorimeter, is attachable to mobile devices, and is controlled by mobile applications [17]. Although more research on those devices should be performed; it shines as a promising innovation. On the other hand, the application of photo cameras and smartphone cameras to determine the color seems more practical since most clinicians already own one of these tools. However, if they are used, it is also necessary to have a computer color matching system to process the image. Fortunately, these systems are becoming as adequate as conventional devices [17,48]. Nonetheless, using these types of devices requires experience in dental photography to obtain the most accurate results. The results may also depend on the mobile phone or camera used. What is more, it should be remembered that in dentistry, macro lenses are usually necessary to capture all the details [16]. The photos are supposed to be of high quality, with appropriate lighting, and without shadow, since shade matching mobile applications use mobile phones as the only device [40]. This poses some limitations, as it requires training in taking appropriate pictures. Such results require trained practitioners with experience in dental photography, as wrongly made photos may result in bias. More research needs to be conducted when it comes to this method of color determination. Mobile applications can be another way to determine and personalize color in prosthodontics [40]. They are easy to use in daily dental practice; it is enough to have a mobile phone. Their cost is much lower than that of other devices for color determination. Mobile apps could be an attractive alternative, which enable a smartphone to be connected with an additional colorimetric device for color measuring. Its interface guides the clinician into a grid in which the tooth should be located and thus helps dentists avoid mistakes while capturing the picture [49]. A more compact, simple-to-use device might be a new branch in the field of color determination in dentistry. In double-blind randomized controlled clinical trials [40], clinically, both methods of color determination, a spectrophotometer—Vita Easyshade V—and mobile application named DENTHUE, showed similar results in shade evaluation. Mobile digital applications can turn out to be a reliable method for shade selection; however, they require further research. What is more, there are already attempts towards developing color measurement precisely through photography by creating an AI program to recognize color changes in photos very accurately, both for use in prosthetic [29,31] and restorative dentistry [50,51].

In one of the included studies, it had been found that the shade selection of dental veneers is especially challenging since they are a thin layer of ceramics and are applied in the esthetic area. Moreover, research shows that regular visual methods are not enough to achieve acceptable results. That is why working with demanding patients requires impeccable esthetics, which can only be achieved using specialized devices for color matching [35]. However, multiple shade measurements in such a scenario should be made, as studies indicated that color determination instruments perform better in full ceramic restorations than in typical esthetic restorations.

A possibly new and interesting approach to ceramic color determination would be intraoral scanners. However, only some of them have a scientifically documented shade measurement function, such as Trios3 (3Shape). In Czigola’s research, the intraoral scanner is said to be a device providing clinically acceptable but not perfect results when it comes to shade selection. This seems to be a promising possibility, considering the increase in the popularity of intraoral scanners, because one device can serve multiple purposes [52]. As for mobile applications, clinicians are more likely to invest in a device that is useful in different clinical aspects. An intraoral scanner can make a digital scan instead of a regular impression, which is a much easier way to store all date and is better for ecological reasons. The color comparison has an accuracy similar to spectrophotometers and that is why it is an attractive alternative [53,54]. However, Rutkūnas’ study shows that the Trios3 intraoral scanner’s accuracy is not enough to be used as an independent shade matching device. This discrepancy suggests that further research is necessary when it comes to intraoral scanners used for shade matching [55].

In summary, the apparent ongoing development in shade determination methods indicates a growing need among the dental community to personalize treatment in the most objective manner possible, but without the need for excessive investments of time and expense. The authors believe that an objective measurement of future porcelain restorations’ color will become a standard of the everyday clinical workflow. This is due to the ever-increasing demands of patients and, unfortunately, the increasing number of cases in which the aesthetics of the prosthetic restoration are questioned after the dentist and patient have already decided on the shade [56]. However, this will be inextricably linked to the development of mobile photography and fast, affordable, and accurate mobile software, which will eliminate possible errors on the part of the dentist and will enable the easy archiving of a procedure.

The limitations of the present systematic review were the numerous tags that characterized different color measurement methods. For this reason, it was impossible to fit all of them in one search. The other limitations stem from the fact that different ceramic restorations have various optical characteristics, as the thickness and opacity of ceramics may differ between them. It should also be noted that the included studies are based on a sample with a small number of specimens or human subjects, depending on the type of study.

## 5. Conclusions

From presented systematic review, the following conclusions have been drawn:

1. The most common method, the visual method, has lower accuracy and repeatability due to its dependence on numerous factors. Devices like spectrophotometers and colorimeters provide precise, repeatable, and objective measurements, but fail to be widely applied in everyday clinical practice.
2. Clinicians should not rely solely on their senses for shade determination, but should turn to quantitative methods.
3. Colorimetric devices connected to mobile applications are an interesting and useful tool, as they combine the advantages of standard objective methods but reduce the cost of their implementation.
4. Software and apps based on artificial intelligence are emerging as promising tools for shade matching, but being a new and rapidly developing solution, they require further research.

## Figures and Tables

**Table 1:** Characteristics of the included studies.

Authors, Year, Country	Type of Study	Number of Subjects	Devices	Main Findings
R.R. Seghi et al., 1989, United States [26]	In vitro study	12 porcelain discs (A1, A2, A3, A3.5, A4, B1, B2, C1, C2, C4, D2, D4)	spectrophotometer Hardy, spectrophotometer Match-Scan Diano, spectrophotometer Spectrogard, colorimeter CR100 Chroma Meter	The colorimeter had a significantly lower ΔE value (CR > 0.19) than all the spectrophotometers. The colorimeter performed best among all the devices and was the most accurate.
Scott R. Okubo et al., 1999, United States [27]	Cross-sectional study	31 observers (7 dentists, 7 dental laboratory technicians, 17 dental students)	Vita Lumin shade guide, colorimeter Colortron II	The colorimeter correctly matched 50% of the tabs, while visual matching identified 48% correctly. The colorimeter demonstrated 100% repeatability. The Colortron II accuracy was only slightly better than visual matching; however, it performed at a higher quality in the case of repeatability.
Shigemi Ishikawa-Nagai et al., 2005, United States [28]	In vitro study	30 CCM specimens (3 CCM specimens for each of the 10 target shade tabs)	spectrophotometer Crystaleye^®^; Olympus (Tokyo, Japan)	ΔE values less than 3.6 were considered clinically acceptable. For areas more than 2 mm from the gingiva, ΔE was less than 3.6, while for the region up to 2 mm from the gingiva, ΔE was 3.9. The overall performance of the spectrophotometer was clinically acceptable, apart from the gingival area, where it showed unacceptable results.
Alvin C. Wee et al., 2005, United States [29]	Cross-sectional study	9 maxillary central incisors (1 was removed during the study)	Vita Lumin Vacuum/VMK68, Vitapan 3D Master/Omega900, IvoclarChromascop/Classic	Vitapan 3D Master showed the highest accuracy (55%), followed by Chromascop (35%), and finally Vita Lumin Vacuum (20%). The Vitapan 3DMaster/Omega 900 performed better in shade-matching than the Vita LuminVacuum/VMK 68.
Joshua Kristiansen et al., 2011, United States [30]	In vitro study	17 ceramic tabs fabricated based on measurements of 17 human maxillary incisors	spectrophotometer Crystaleye^®^; Olympus (Tokyo, Japan)	The spectrophotometer reproduced the colour of natural teeth with a mean ΔE* of 2.58. This system has the potential to be used clinically for color determination.
Wang, Peng et al., 2014, China [31]	In vitro study with neural network research	32 metal ceramic tabs (27 used for training the BP neural network, 5 for testing the system)	Spectrophotometer, BP neural network model	The precision of the matching among the test group and the forecast of corresponding test tabs was 80%, but the average color difference between them was 1.68, while the clinically tolerable threshold was 2.7. Shade matching employing backpropagating neural networks provides a promising method for shade determination.
Jian Wang et al., 2014, China [32]	In vitro study	21 porcelain discs	CCM system, spectrophotometer Crystaleye (Olympus Corp), VITAPAN 3D-Master shade guide	The ΔE* values among computer color matching specimens and the target shade tabs showed an average ΔE* of 1.3, which was significantly less than the clinically detectable ΔE* threshold of 1.6. The CCM system is effective and accurate in clinical use.
Jiaqiang Wei et al., 2016, China [33]	In vitro study with neural network research	43 metal–ceramic specimens (39 for training the BP neural networks, 4 for testing and calibrating)	spectroradiometer PR-655 Spectra Scan, backpropagation neural network, traditional visual method	The medium ΔE value of the CCM system was 1.89 ± 0.75, which was less than that of the visual approach (3.54 ± 1.11, *p* < 0.01). The CCM system showed a higher accuracy in color reproduction than the visual approach.
Satheesh B. Haralur et al., 2016, Saudi Arabia [34]	Cross-sectional study	Two porcelain tabs fused to metal discs	Myers–Briggs Type Indicator (MBTI), a questionnaire that distinguishes different personalities	The personalities that performed best in tooth shade selection were ENTJ (extraversion/intuition/thinking/judging) (2.923 ± 2.36), ISTJ (introversion/sensing/thinking/judging) (3.086 ± 2.56), ENFJ (extraversion/intuition/feeling/judging) (3.197 ± 2.936), and ESTJ (extraversion/sensing/thinking/judging) (3.431 ± 2.78). There was a statistically significant difference among the different personalities considering visual color matching.
Mohammed. S. Bin-Shuwaish et al., 2021, Saudi Arabia [35]	In vitro study	32 dental laboratories (16 government and 16 commercial), 32 porcelain crowns fused to metal crowns	Comparison of thirty-two dental labs	The quality of marginal adaptation of crowns was good in 81.25%; however, the quality of contours, contacts, and shade matching was compromised in 43.75%, 59.38%, and 39% of all labs, respectively. Visual and colorimeter shade matching was acceptable in 62.5% and 80% of labs in the cervical third and middle third regions of crowns, respectively; however, in the incisal third, the shade matching was unacceptable in nearly 60% of labs. Commercial laboratories presented significantly better contours and shade matching, but not marginal adaptation.
Xue-Dong Bai et al., 2021, China [36]	In vitro	Eighteen veneer discs (shade A2 and 0.7 mm in thickness) were fabricated using 6 veneer materials. The veneer specimens placed on 5 extracted teeth with nominal shade A2 formed veneer–tooth combinations.	Vita Lumin Vacuum, a spectrophotometer (PR-655 SpectraScan, equipped with MS-75 and a SL-0.5X lens	The ΔE of the veneer–tooth combination was on average 3.1833 ± 1.5485. Mean of ΔE of veneer–tooth combinations to the shade tab was 4.0103 ± 1.8508. Translucency parameter values decreased gradually as the veneers became thinner. When the thickness of veneers is lower than 0.7 mm, the visual shade replica protocols are not enough for acceptable results.
G.E. Adebayo et al., 2022, Nigeria [37]	Cross-sectional study	24 patients with 26 teeth, 26 porcelain fused to metal crowns	three categories of dental professionals; a specialist restorative dentist (consultant), a dental surgery intern (house officer) and a dental surgery technician (dental nurse), VITA classical shade guide	The specialist restorative dentist matched the tooth shade with the spectrophotometer in 11.5% of cases, which was the best result among all dental professionals. Inter-examiner reliability was very low. Experience and training in shade selection might play a role in correct selection.
Mohammed A. Akl et al., 2022, United States [38]	In vitro study	16 specimens in the form of metal–ceramic restorations	VitaEasyshade, Spectroshade, Spectroradiometer PR670	When comparing the mean ΔE00, the differences in values between PR670 and Spectroshade were not clinically significant. Spectroradiometer PR670 is the gold standard in color matching. The SpectroShade instrument can be of great benefit for both shade matching and color research.
Garg, Anirudh et al., 2022, Germany [39]	Cross-sectional study	5 tabs (A2, A3.5, B1, C2, and D3), 104 participants (under 30 years of age) were divided into two sets (52 participants per group) based on their clinical experience	Vitapan Classical shade guide	Correct shade matching with the monocular dominant vision was 53%, which was significantly better compared to the monocular non-dominant vision at 12% or binocular vision at 44%. Clinical experience has shown that there is no significant difference in visual shade matching. Sex showed a slight influence in shade matching, with higher accuracy for women. Ocular dominance played a significant role in the accuracy of visual shade selection.
Chaware, Sachin Haribhau et al., 2023, India [40]	Randomized clinical trial	30 participants evaluating 45 full-coverage metal–ceramic restorations and 45 full-coverage all-ceramic restorations	Vita 3D Master shade guide, Vita Easyshade spectrophotometer, and mobile application for each participant	ΔE values of Vita Easyshade and the mobile digital application were similar. The mobile phone application might appear as a reliable method for shade selection.
Chih-Te Liu et al., 2023, Taiwan [41]	In vitro study	6 porcelain veneers	AUO Display Plus, Canon single-lens reflex camera with eLAB’s polar eyes filter, VITA Easyshade V	The ΔE of the AUO Display Plus had the smallest differences between the color of the fabricated teeth and that of the original teeth. Advanced Reflectionless Technology, provided in AUO Display Plus, is better than traditional monitors.

**Table 2:** Newcastle–Ottawa Scale adapted for cross-sectional studies.

Author		Scott R. Okubo et al., 1999 [27]	Alvin C. Wee et al., 2005 [29]	Satheesh B. Haralur et al., 2016 [34]	G.E. Adebayo et al., 2022 [37]	Garg, Anirudh et al., 2022 [39]
Selection: (Maximum 5 stars)	(1) Representativeness of the sample	*		*	*	*
	(2) Sample size					*
	(3) Non-respondents			*		*
	(4) Ascertainment of the exposure (risk factor)	*	*	*	*	*
Comparability: (Maximum 2 stars)	(5) The subjects in different outcome groups are comparable, based on the study design or analysis. Confounding factors are controlled.	**	*	**	**	**
Outcome: (Maximum 3 stars)	(6) Assessment of the outcome **	**	**	**	**	**
	(7) Statistical test	*	*	*	*	*
	Total score=	7	5	8	7	10

**Table 3:** Evaluation of in vitro studies according to the QUIN assessment tool.

Criteria No.	Criteria	R.R. Seghi et al., 1989 [26]	Shigemi Ishikawa-Nagai et al., 2005 [28]	Joshua Kristiansen et al., 2011 [30]	Wang, Peng et al., 2014 [31]	Jian Wang et al., 2014 [32]	Mohammed. S. Bin-Shuwaish et al., 2021 [35]	Xue-Dong Bai et al., 2021 [36]	Mohammed A. Akl et al., 2022 [38]	Chih-Te Liu et al., 2023 [41]
1	Clearly stated aims/objectives	2	2	2	2	2	2	2	2	2
2	Detailed explanation of the sample size calculation	0	0	0	0	0	0	0	0	0
3	Detailed explanation of the sampling technique	2	2	2	2	2	2	2	2	2
4	Details of the comparison group	2	2	2	2	2	2	2	2	2
5	Detailed explanation of methodology	2	2	2	2	2	2	2	2	2
6	Operator details	2	2	2	2	2	2	2	2	2
7	Randomization	0	0	1	0	0	2	0	2	2
8	Method of the measurement of outcomes	2	2	2	2	2	2	2	2	2
9	Outcome assessor details	0	0	1	0	0	1	0	0	0
10	Blinding	0	0	2	0	0	1	0	0	0
11	Statistical analysis	2	2	2	2	2	2	2	2	2
12	Presentation of results	2	2	2	2	2	2	2	2	2
13	Overall score	Medium	Medium	High	Medium	Medium	High	Medium	Medium	Medium

**Table 4:** Jadad scale for reporting randomized controlled trials.

Author	Chaware, Sachin Haribhau et al., 2023 [40]
(1) Is the study described as randomized?	*
(2) Is the study described as double blind?	*
(3) Is there a description of withdrawals and dropouts?	*
(4) The method of randomisation is appropriate?	*
(5) The method of blinding is appropriate?	*
Total score=	5

*—one point.

## Data Availability

Data are available from the corresponding author upon reasonable request.

## Supplementary Materials

The following supporting information can be downloaded at: https://www.mdpi.com/article/10.3390/jpm14030252/s1, Supplementary Materials S1—Prisma 2020 Checklist.
